# Unconventional therapy use among asthma patients in a tertiary care center in Riyadh, Saudi Arabia

**DOI:** 10.4103/1817-1737.39636

**Published:** 2008

**Authors:** Mohamed S Al Moamary

**Affiliations:** *College of Medicine, King Saud bin Abdulaziz University for Health Sciences and Pulmonary Section, King Abdulaziz Medical City, Riyadh, Saudi Arabia*

**Keywords:** Asthma, Saudi Arabia, uncontrolled asthma, unconventional therapy

## Abstract

**OBJECTIVES:**

Unconventional therapy (UT) is a therapeutic practice of alternative and complementary medicine that is not currently considered an integral part of modern medical practice. The aim of this article is to investigate the experience of Saudi patients with UT modalities in the treatment of asthma.

**MATERIALS AND METHODS:**

We carried out a cross-sectional study of asthma patients referred to King Abdulaziz Medical City, Riyadh, Saudi Arabia, during the year 2004. Information was collected using a pre-designed questionnaire administered through interviews.

**RESULTS:**

Two hundred consecutive patients with a mean age of 52.3 years (±18.7) were included in this study. Sixty-nine (34.5%) of those patients used some form of UT in the previous year. There was a tendency to use UT among the older age group (*P* = 0.029) and among those with longer duration of disease (*P* = 0.009). However, there was no significant correlation observed between the use of UT and gender, FEV_1_, or disease control. The most commonly used form of UT was recitation of Holy Quran (9%), honey (24.5%), herbs (23.5%), cautery (12%), and blackseed (10%). There was no significant correlation between disease control and the use of modalities.

**CONCLUSION:**

Unconventional therapy is frequently practiced by asthma patients in Saudi Arabia, who commonly believe that UT will lead to improvement. The lack of evidence necessitates the fostering of a national project to address the practice of UT.

Asthma is a chronic inflammatory disorder with airway hyper-responsiveness that leads to recurrent episodes of wheezing, breathlessness, chest tightness, and coughing. These episodes are usually associated with reversible airflow obstruction.[1] Modern guidelines for asthma management are based on the treatment of the inflammatory process and the bronchoconstriction by anti-inflammatory agents and bronchodilators respectively.[2] To date, there is no approved curative treatment for asthma.[1] This has led to considerable individual health care expenditure, which included the widespread use of unconventional therapy (UT) in the hope for a cure.[3–4] UT has evolved over thousands of years with transmission of experiences among different cultures. It includes complementary and alternative medicine and has been defined as the diagnosis and/or prevention which complements mainstream medicine by satisfying a demand not met by modern medicine.[5] In western countries, 42 to 59% of asthma patients have used alternative medicine during the course of their illness; while[5–7] other countries, there has been a move toward using various traditional methods of healing.[5–6] The most popular alternative medical treatments are herbs (western and Asiatic), acupuncture, various types of body manipulations, psychological therapies, and homeopathy.[3] These modalities of treatment cannot be recommended for asthma management since the therapeutic and psychotherapeutic benefits of unconventional therapy have not been validated by conventional standards and are difficult to evaluate in randomized controlled trials.

Saudi Arabia is a country that has invested heavily in the infrastructure of a modern health care system in the past three to four decades. The world map developed by the World Health Organization shows that more than 95% of the Saudi population has access to asthma medications.[8] Nevertheless, it has been observed that many Saudi patients still use unconventional modalities during the course of their treatment. To the best of author's knowledge, no studies to address this issue have been conducted in Saudi Arabia. Therefore, the aim of this paper was to investigate the nature, prevalence, associations, and experience of Saudi patients who use unconventional modalities to treat their asthma.

## Materials and Methods

A cross-sectional study was carried out on patients diagnosed with asthma who were referred to the Adult Pulmonary Clinic during the year 2004 at King Abdulaziz Medical City, Riyadh, Saudi Arabia. The inclusion criteria included age above 21 years, history of asthma for more than a year, and consenting to answer the study questionnaire. The patients were interviewed using a pre-designed questionnaire that included demographic data, compliance to medications, and the use of unconventional modalities in the previous year. Disease severity was determined by the application of the Global Initiative for Asthma (GINA) classification of disease severity.[2] Data were also analyzed based on the newly released disease control scale by GINA.[9] Forced expiratory volume in one second (FEV_1_) was done by Jaeger Master Lab, Germany. FEV_1_ was calculated from the best of three trials according to the criteria recommended by the American Thoracic Society.[10] The association between the use of UT and different patient characteristics was calculated. After verbal consent, patients were interviewed during their session for asthma education. It was emphasized that this information would remain confidential and would be utilized for study purposes only.

### Statistical analysis

Continuous variables were summarized by calculating the mean and the standard deviation, whereas categorical variables were summarized by calculating the number and percentage. The association between the use of UT and the different categorical variables was assessed by using the Chi-squared test (or Fisher's exact test, as appropriate), whereas the t-test was used for the association with continuous variables by using SPSS program.

## Results

**Figure 1:**
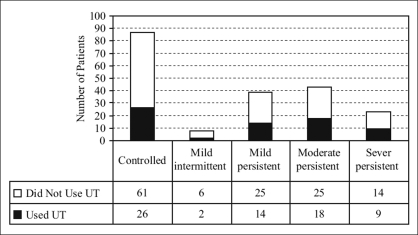
Use of unconventional therapy among asthma patients categorized based on disease severity index. *UT: Unconventional therapy*

Two hundred consecutive patients were interviewed. The mean age was 52.3 years (±18.7). There were 78 males and 122 females, with male:female ratio of 1:1.56. The educational level was more than the high school level in 113 patients (56.5%). The percentage of mean FEV_1_ within 12 months prior to the interview was 74% (±28.7%). Sixty-nine (34.5%) patients used unconventional therapy. Figure 1 shows the frequency of UT usage categorized on the basis of disease severity. There was no significant correlation observed between the use of UT and disease severity (*P* = 0.66).

Twenty-seven (34.2%) male patients used UT to 42 (34.7%) female patients, which was not significant (*P* value = 0.938). There was a tendency to use UT among the older age group with mean age of 56.2 years (±18.7), compared to mean age of 50.2 (±18.2) for those who did not attempt UT (*P* = 0.029). There was also a trend of using UT in asthma patients with longer duration of disease, with a mean duration of 16.0 months (±12.2), compared to mean duration of 11.2 (±11.9) for those who did not attempt UT (*P* = 0.009). The mean percentage of FEV_1_ was 69.8% (±29.7%) for those who used UT, compared to 76.1% (±28.1) for those who did not use UT, which did not reach statistical significance (*P* = 0.15). Fifty (44.3%) patients with educational level more than the high school level used UT, compared to 19 (21.9%) patients with educational level less than the high school level who used UT (*P* < 0.001).

Table 1 shows the most frequently used UT modalities in the study population. Of the 69 patients who used UT, 31 (44.9%) used more than one modality. The following modalities were used in less than 1% of the study population: caffeine, ginger (*Zingiber officinale*), garden cress (*Lepidium sativum, Arabic: Rashaad*), acupuncture, tea, and mint.[11–12] There was no significant association between disease control and the use of UT, which included Holy Quran recitations, honey, herbs, and cautery [Table 2].

**Table 1:** Most frequently used UT modalities in 69 patients

Modality of unconventional therapy	No.	Percentage
Holy Qur'an recitations	18	9.0
Honey	49	24.5
Herbs (not specified)	47	23.5
Cautery	24	12
Black seeds (*Nigella sativa*), (*Arabic: habbah Albarakah*)	20	10.0
Myrrh (*Commiphora myrrha*), (*Arabic: murra*)	14	7.0
Garlic (*Allium sativum*)	9	4.5
Gum (not specified)	7	3.5
Fenugreek (*Trigonella foenum-graecum*), (*Arabic: hulba*)	7	3.5

**Table 2:** The correlation between frequently used UT modalities and disease control

Modality	Usage among controlled patients (*n* = 28)	Usage among partially or un-controlled patients (*n* = 39)	*P*-value
Holy Qur'an	2 (7.1)	8 (20.5)	0.13
Honey	17 (60.7)	29 (72.5)	0.31
Herbs	18 (64.3)	27 (67.5)	0.73
Cautery	6 (21.4)	15 (37.4)	0.16

Figures in parentheses are in percentage

**Figure 2:**
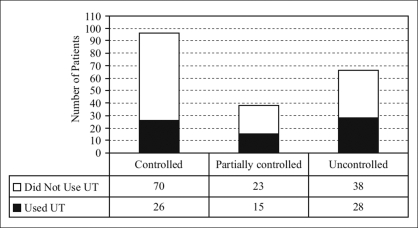
Use of unconventional therapy among asthma patients categorized based on disease control index. *UT: Unconventional therapy*

By applying the newly introduced disease control index, 96 (48.0%) patients had controlled disease, 38 (19%) had partially controlled disease, and 66 (33%) had uncontrolled disease. Figure 2 shows the frequency of UT among asthma patients categorized based on disease control index. There was no significant correlation observed between use of UT and disease control (*P* = 0.1).

Out of the 69 patients who used UT, 39 (57.0%) claimed to have observed some benefits; out of whom 26 (66.7%) would advise their friends to try UT. Nevertheless, 59 (85.5%) believed that modern therapy was more effective than UT. Most of the study population (98.5%) continued to use conventional medical treatment, including those who were trying UT.

## Discussion

Unconventional therapy has grown considerably in the past decades despite the limited evidence of the efficacy of most UT modalities.[13] The increasing interest in UT on the part of media has led to more patients being aware of alternative methods of asthma treatment. A report from Switzerland has estimated considerable cost of covering common alternative methods by health insurance.[14]

The favored conventional drugs used in the second half of the 20^th^ century had their origins in folk medicine and were developed by isolating the active substances or duplicating them in the form of synthetic products.[15] Ephedrine was developed from the Chinese herb *ma huang.* Theophylline was found in tea leaves. An anticholinergic effect was achieved by heating henbane leaves. Cromolyn, which is a derivative of *Ammi visnaga,* is the source of the bronchodilator Khella, found in the Middle East.[16,17]

In this study, approximately one-third of the study population had tried UT. In the United Kingdom, 60% of people with moderate asthma and 70% with severe asthma have used complementary or alternative medicine.[5] Another survey from South Australia showed that 55% of children used at least one form of UT.[18] In Saudi Arabia, the use of UT has been reported in 18 to 30% of patients with chronic conditions.[19–22] In this study, 57% reported observing some benefits from UT. Although two-thirds would advise their friends to use UT, 85% would continue to use modern medications - an interesting finding that could indicate that the observed improvement from UT is subjective. Another explanation for the observed benefits is related to the placebo effect from the UT, since most of our patients continued to use UT with their regular medication.[23] This makes it difficult to attribute the observed benefits to UT alone. On the other hand, asthma is an episodic disease in which a patient can improve with or without medications. These concerns are not meant to downgrade UT but should be addressed by collecting further evidence to determine the risks *vs.* the benefits of UT.

The author has observed that Holy Quran recitations are frequently practiced for healing purposes in Saudi Arabia, which is related to the deep belief prevailing in the studied community that *{If Allah touch thee with affliction, none can remove it but He; if He touch thee with happiness, He hath power over all things}* (Quran; Sura no. 6: verse no. 17). There are several verses in the Holy Quran related to the healing effect *{O mankind!, there hath come to you an admonition from your Lord and a healing for the (diseases) in your hearts and for those who believe, a Guidance and a Mercy} (Quran; Sura 10: verse 57).* This verse explains why the believers would ask for Quran recitation to heal their disease.

One-quarter of the study population used honey as UT for asthma. To the best of author's knowledge, there is no available scientific evidence yet to support the use of honey in asthma.[24]

Ten percent of the study population used blackseed. “*There is healing in the blackseed for every disease except death*,” prophet Mohammed said over fourteen centuries ago. Ibn Sina (980–1037), in describing the blackseed, said, “*It stimulates the body's energy and helps recovery from fatigue or disspiritednessy*.” A report from Saudi Arabia showed that blackseed has thymoquinoneinduced relaxation of guinea-pig isolated trachea, either alone or in combination with honey.[25] There is also an evidence suggesting that there is subjective improvement in asthma patients through bronchodilator effect.[26–27]

Garlic, which was used in 4.5% of the patient population, has some physiological value in that it improves natural body defenses and may be beneficial to chronic airway inflammation.[6] In a Cochrane review update in 2001, it was concluded that caffeine appears to improve airway function modestly in people with asthma for up to 4 h.[28] Caffeine is chemically related to theophylline and is considered as a weak bronchodilator.[29] Despite this scientific evidence, coffee and tea were consumed by a minority of the study population. To the best of author's knowledge, there is no available evidence for objective improvement based on measured physiological parameters for the previously mentioned UT modalities. Almost a quarter of the study population used local herbs from local perfume makers (*Arabic: attar*). These were given as unknown mixtures to the patients. The contents of such mixtures raise genuine concerns since some perfume makers may mix asthma medications such as theophylline and corticosteroids with herbs.[30] In a national survey conducted in the United Kingdom, herbal remedies were the third most popular choice among UTs for asthma.[31] In a systemic review of the usage of different mixtures of herbal remedies for asthma, no definitive evidence for any of the reviewed herbal preparations emerged to be of clinical value. Some of the other forms of UT that were used in other communities were not available and subsequently not used by this study population, e.g., acupuncture, homoeopathy, and breathing techniques.

Some limitations should be noted with regard to this study. The patients represented those referred to a tertiary care center, where more patients are expected with moderate and severe disease. Also this sample might not represent the pattern of all UT modalities used in the community, as some patients may have felt reluctant to disclose to a health care professional the type of UT that they used.

Unconventional therapy is frequently practiced by patients with asthma in Saudi Arabia who hold the common belief that it will lead to improvements. This would lead to certain implications from this study: (1) it is recommended that the use of UT undergo further investigation to evaluate the extent of the use and efficacy; (2) it would be interesting to conduct randomized controlled studies to obtain evidence for the benefits of UT and to evaluate the positive perception that patients have observed form UT; (3) because individuals using UT are more involved in the decision-making process and have more control of the illness, they would attempt to use these modalities.[32] This necessitates that health care workers dealing with asthma cases be informed about the different modalities of UT in order to provide appropriate information to the patients; (4) with currently available evidence, physicians dealing with asthma cases cannot recommend UT. Physicians should encourage their patients to disclose openly any use of UT.
